# An evidence-based model to promote community engagement in health intervention research: the Quiet 4 Healthy Farm experience

**DOI:** 10.3389/fpubh.2025.1684720

**Published:** 2025-09-30

**Authors:** Marjorie C. McCullagh

**Affiliations:** Department of Systems, Populations, and Leadership, School of Nursing, University of Michigan, Ann Arbor, MI, United States

**Keywords:** community engagement, model, clinical trial, recruitment, retention

## Abstract

Community engagement involves working collaboratively with groups of people on issues affecting their well-being. The process is central to public health. Although the concept of community engagement is based on the assumption that active participation by community residents in the process of improving health and social outcomes will lead to an empowered community, and long-term health and social improvements, there is little research on how to achieve community engagement. This article describes a variety of the author’s practices in community engagement in the context of community-based health promotion intervention research. The practices highlighted in this perspective are highly adaptable to other studies, participants, settings, and health issues, and have potential for augmenting the community engagement and success of future community-based health behavior studies, and consequently contribute to the public health.

## Introduction

The Centers for Disease Control (CDC) has defined the term community engagement as “the process of working collaboratively with groups of people who are affiliated by geographic proximity, special interests or similar situations with respect to issues affecting their well-being.” The CDC describes community engagement as a process extending from reaching out to community members (outreach), to consulting with them (consultation), to collaborating to develop shared goals and implement activities to address community needs (collaboration). However, renowned community-builder Peter Block (1) expresses it eloquently writing, “Community engagement is the art of creating a place of belonging, for it is through engagement that we discover and deepen our connection to one another” Public health seeks to use this community-building to positively affect the well-being of the community.

Community engagement is of great importance to public health research in that it is strongly associated with intervention effectiveness. Multiple studies have shown that community engagement interventions have a positive impact in the context of a variety of populations, settings, and health outcomes (2–4). Additionally, maintaining participant engagement in the form of retention is crucial for preserving the statistical power and internal validity of longitudinal studies. When attrition rates are high, there is a greater chance of introducing bias, especially if those who drop out differ from those who remain, or if attrition rates vary between the intervention and control groups in a randomized controlled trial (5) However, the models and methods of community engagement vary widely across studies, and there is little evidence that any one model or method is more effective than others. Further, the literature lacks consensus on methods, best practices, and measurable outcomes for community engagement.

The author developed a model of community engagement based on research experience (6–8) aimed at increasing use of hearing conservation behaviors among noise-exposed agricultural workers. The Quiet 4 Healthy Farm suite of intervention studies focuses on prevention of noise-induced hearing loss, a highly prevalent but preventable problem among farmers and farm family members (9).

## Barriers to community engagement

Researchers in these studies experienced multiple significant barriers to involvement in research among individuals and groups in this population. The number of farming operations in the US is about 2 million (US Department of Agriculture National Agricultural Statistics Service (10)), and the operations are geographically dispersed and not organized by any large entities (11, 12). In addition, farmers have a unique identity and culture; for example, seasonal variations in work hours and tasks, and often-extended workdays (11). Most US farms are family-owned and small (10), giving the farmer and farm family great agency in the operation. Farm operators are characteristically independent and strongly protective of their operational autonomy (13). Farmers lack the support to hearing health typically offered to workers in the manufacturing sector [e.g., noise surveillance, hearing protection, hearing conservation education, and related supervision (14)]. Further, farm operators are considered a hard-to-reach population in terms of epidemiological and intervention research (15).

Retaining individual and organizational engagement in research is an ever-present challenge of longitudinal studies (16). When attrition rates are high, there is a greater chance of introducing bias, especially if those who drop out differ from those who remain, or if attrition rates vary between the intervention and control groups in a randomized controlled trial (5) A number of factors present obstacles to engagement between the community members (as individuals and community groups) and the research team.

The purpose of this report is to present the author’s model of community engagement. This model may be used to form the foundation for the development of measures of community engagement relevant to delivering community health promotion interventions and improving the health of the public. The methods described address community engagement at two levels: individual study participants, and groups.

## Research overview

The model described here is primarily based on two community-based health behavior intervention studies conducted between 2010 and 2020. One of the studies was a randomized controlled trial of 491 farm operators assigned to one of five intervention groups (17). A second study of focus was a cluster randomized control study of farm and rural youth comparing the effectiveness of two interventions and a no-interaction control (18).

Individual participants in the farm operator study included a convenience sample of farm operators from the US and Canada. Group participants included farm organizations (e.g., commodity and advocacy groups). Participation was through mail, email, Web site, telephone, and individual and group face-to-face sessions. Individual participants in the farm youth study included English speaking students enrolled in grade four who attended any one of 36-community based educational events in the US sponsored by a partner organization (i.e., Progressive Agriculture Safety Day).

## Community engagement model

**Figure 1:**
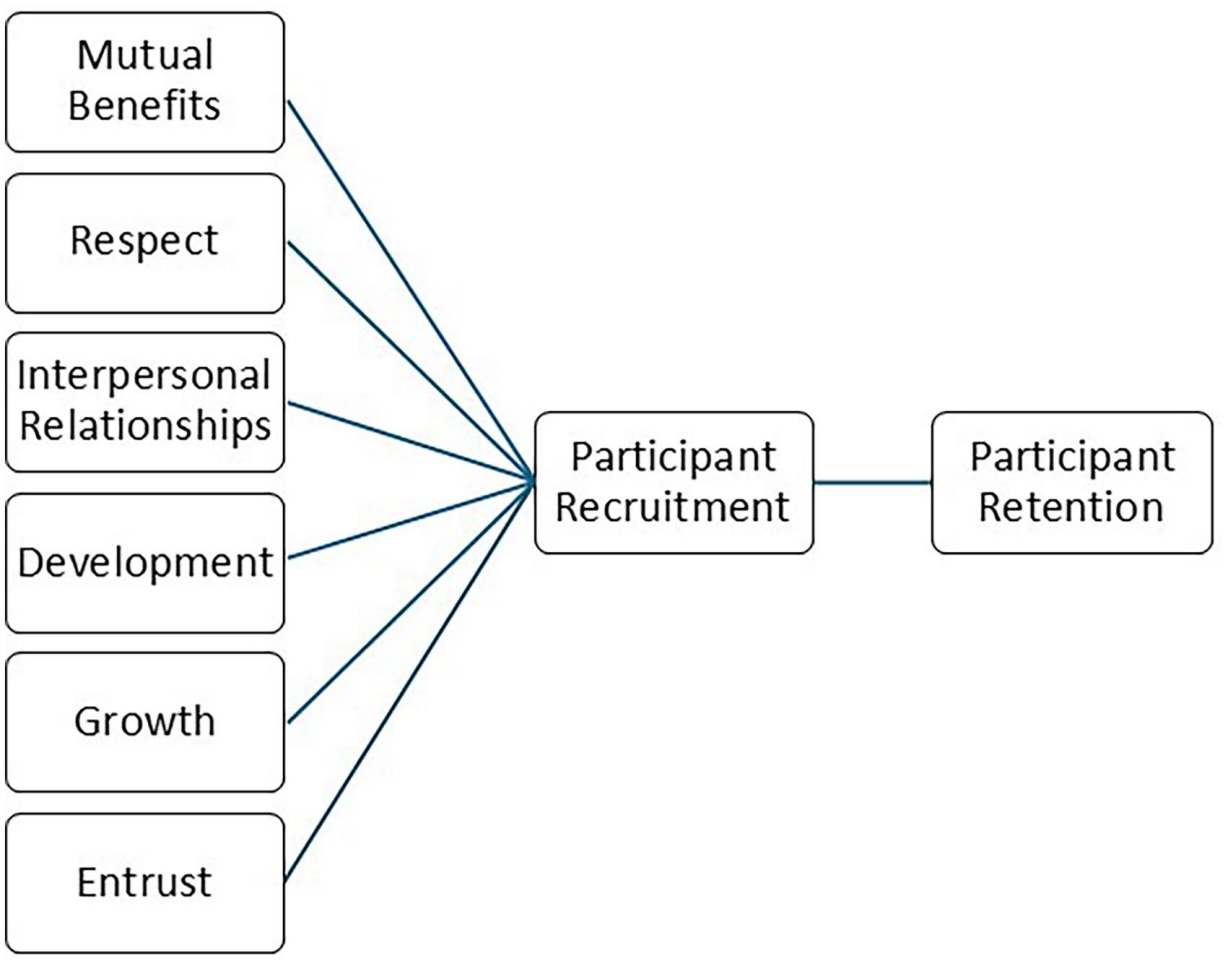
BRIDGES model.

Working closely with a highly dedicated and skilled team of researchers engaged in a community-based health promotion intervention program of research, the author employed a variety of community engagement practices, described here and represented by the acronym *BRIDGE:* mutual Benefits, Respect, Interpersonal relationships, Development, Growth, and Entrust (Figure 1). Together, these concepts were applied in the form of strategies of interaction with actual and potential research participants by research team members in the context of a community-based clinical trial. Each of the concepts (and associated strategies) is positively associated with the defined outcomes of participant recruitment, and in turn, retention. Together, the concepts and associated strategies were designed to promote successful participant recruitment and retention, thereby supporting accomplishment of study aims.

### Mutual benefits

The study team was intentional in creating a system of mutual benefits. In the context of this model, benefits are things that is good or helpful from the perspective of the study partncipant at the individual or organizational level. These benefits took the form of structured incentives at the individual or organizational level. For example, the study offered benefits for farmers at both the individual and organizational levels. For example, partner organizations were selected for participation based on their espoused commitment to farmer health and safety. These organizations connected with and built on existing strengths and resources, including existing safety personnel and programs, large numbers of members or participants, an established organizational hierarchy, and existing media (e.g., Web, print) for communication with members. The team identified gatekeepers within each organization, creating liaisons and cultural brokers between the study team and study participants. These organizations benefited from the opportunity to offer free hearing health programming for their members, while individual farmers were attracted to associated benefits, such as new information that related to their personal safety and health, as well as an opportunity to collect incentives in the form of cash and supplies of personal protective equipment for their personal use.

The investigating team sought engagement with community organizations whose missions were consistent with the goals of the intervention program, thus promoting benefits for both partners. Groups approached for participation included commodity groups, advocacy organizations, and education organizations. In addition, funding for the intervention studies was obtained from government-sponsored research organizations as well as a for-profit commercial entity with an interest in personal protective equipment sales.

Further, the study team used a variety of financial and other incentives to motivate individual participants to enroll and complete the study and employed recruitment messages to appeal to potential participants’ diverse motivations for enrolling and remaining in the study through a full year of data collection. The study team negotiated individually with a representative of each farm organization to develop strategies for working with the organization. In one of the studies, a four-part system of incentives was employed to address a variety of influences on study participation. First, individual study participants were eligible to receive a cash award at completion of each of three waves of data collection. These individual participant incentives were graduated ($10 at wave 1, $10 at wave 2, and $20 at wave 3).

Aside from individual participant incentives, a separate incentive strategy was offered to participating organizations. The study team contracted with farm organizations to award organizations that successfully met participant study completion goals. The study team leveraged this organizational incentive to encourage individual study participants to use their participation to earn credits for their respective organization.

Study participants were informed that study participation involved resources that could help them to better understand hearing hazards on their farm, and to self-select measures to protect their hearing. Additionally, study participants were reminded that results of the study would be used to develop programs to help other farm operators. In this way, the study team leveraged multiple potential motivators for potential participants to engage in the study: personal financial, organizational financial, personal safety, and altruism.

The study also incorporated a focus on mutual knowledge building in two ways. First, the study team promoted the concept that the farmers were the experts in farm-related noise exposure and use of hearing protection devices. The study team was described as the knowledge-seekers in the context of farmers’ participation. The study team emphasized that individual farmers, the participating farm organization, and the community of farmers as a whole would all benefit from the knowledge gained through farmer participation, further tapping into prospective participants’ altruism.

### Respect

The team demonstrated primacy of respect for the study participant in all contexts. In the context of the proposed model, respect is actions demonstrating consideration and deference to the preferences and needs of study participants. Respect took the form of minimizing us participants’ time and protecting their privacy. For example, the study coordinator was faithful in promptly delivering study materials and incentives to participants. When reaching out to nonrespondents was necessary, study team members used the participants expressed preferred method (e.g., email, telephone), but limited contacts to a small number and during times of the day to minimize interruption in family and rest time. Study recruiters repeated contacts to individuals expressing interest in the study no more than twice. To avoid the appearance of harassing or bothering prospective enrollees, study team members employed sensitivity and discretion in soliciting attendees to enroll in the study.

Further, the study team worked to enhance the convenience of participation among farm organizations. Study personnel used personal contacts (rather than electronic methods) whenever possible to communicate with farm organization representatives, and emphasized to farm organization staff the relevance of study aims to their farm organization mission. The team also worked with each farm organization to individually tailor an informational program for their members. For example, the team offered one-on-one conversations at farmer organization meetings, group presentations at farm organization staff meetings and webinars, and assistance in preparing newsletter and Web communications. The study team developed a variety of electronic, print, and visual messages that were designed to appeal to an array of prospective study participants, and logo branding of the project was included on study messages. These actions were attractive to farm organizations in that they increased capacity to provide member services. Concurrently, researchers obtained valued access to members of this hard-to-reach population.

### Interpersonal relationships

The study team used long-standing interpersonal relationships and proven interpersonal interaction strategies to foster community engagement. In the context of the proposed model, interpersonal relationships are characterized by social connections with study participants. These social connections were characterized by mutual support and minimization of power differentials between participant and researcher. The research projects were based on long-standing partnerships between the principal investigator and a major farmer advocacy organization. Having established a long history of engagement with this population, the farm population had already demonstrated their favorable attitude toward health and safety research in previous studies with this investigator. The investigator used this prior experience to employ recruitment strategies developed and tested with similar groups.

The study team designed the program to engage members of the farming community in the project from the earliest phases of project development in the form of advisors, panelists, and beta testers. For example, the study team worked individually with each local organization to develop marketing and participation plans specific to that organization.

### Contextually appropriate for developmental level

The study was designed to engage farm workers and their families in ways that were relevant to their lives. In the context of the proposed model, development is considered activities focused on improving participants’ lives. These actives included expanding their opportunities and capabilities for long and healthy lives at work and beyond, as well as being knowledgeable about their work- and recreationally-related noise hazards, and optimizing their quality of life. Suggested intervention strategies were targeted to the unique work tasks and lifestyles of this worker group. The language level of written and oral messages was consistent with the typical educational level of this group. Posters, handouts, and Web-based images featured workers, tasks, and equipment that were commonly found in the farm work setting. Barriers to use of health-promoting behaviors specific to the farm operation were addressed, and strategies recommended by experienced farmers to increase use of hearing protectors were highlighted.

While securing the initial engagement of study participants was a challenge, retaining their engagement over a period of the many months of the study was even more challenging. Personal contact with study participants using multiple follow-up methods, often by telephone, proved to be one of the most effective methods of retaining participant engagement. This approach was challenging in that it was labor-intensive, not practical for most hours of the day during the growing and harvesting seasons, and difficult to implement in the multiple time zones represented in the study population.

### Growth through cyclical and iterative refinement of community engagement strategies

Throughout the recruitment and retention phases of the study, the study team was vigilant in monitoring and evaluating participant engagement. In the context of the proposed model, growth is considered change that favors achievement of program aims. Changes included planned interventions and other study-related activities, such as communication with study partners. The team continuously tracked subjects’ follow-up status and completion rates, providing the team with real-time data on the effectiveness of their engagement strategies, and offering opportunities to modify recruitment and retention approaches. For example, although the team was advised that a slow recruitment pattern was common and enrollment could be expected to increase, recruitment data did not bear this out and the team consequently rejected this advice and proceeded with altering the recruitment plan. In addition, the team trialed external supports in the form of a commercial email list service and expert research consultants. However, enrollment data again indicated these strategies were not effective, and the team generated alternative strategies that proved effective.

Although meeting enrollment targets was necessary for study success, completion of 12-month follow-up surveys (rather than enrollment numbers) served as the ultimate goal for the study team. The team monitored survey completion rates at frequent intervals, and results were used to inform engagement methods. When study data revealed that a substantial number of participants were not responding to an automated 6-month email reminder to submit survey data, the team again responded with an adjustment to study participant engagement strategies. The team determined that these automated reminder emails from the study were inadvertently diverted to participant junk email boxes. To counteract this obstacle, the team sent additional reminder (“booster”) emails to each non-responding participant, which proved effective. Further, evaluation data showed that some participants were slow to access the study Web site, which the team determined was related to either poor computer literacy or poor Internet access in their rural communities. Consequently, the study team initiated telephone contacts with participants, offering a non-computer-based alternative for completing data collection via telephone. Additionally, it became apparent that relocation was another contributor to participant attrition. The study team initiated postal reminders and Web-based searches were used to locate more mobile study participants.

Study team members encouraged snowballing as a further engagement method. In this way, pre-existing relationships between community members were used to share study information with friends and/or family members who might be interested.

### Entrust

The study team sought endorsement from a variety of farm organizations, thus promoting trust among members of these organizations who were prospective enrollees. In the context of the proposed model, entrust is a belief (among actual and potential study participants in the reliability and ability of the research team to promote the welfare of study participants. This required a focus on development of trust between individual study participants (actual and potential) as well as participating organizations. Although individual incentives were offered to participants (e.g., free hearing protection devices), many farmers were motivated to participate because they trusted that the knowledge gained by the research study would benefit other farmers in the near and distant future. Each of the intervention studies engaged multiple organization partnerships. For each partnership, the research team tailored a unique engagement plan specific to the organization’s mission, membership, and organizational structure. Often, the team was invited to attend farmer commodity and advocacy group meetings. Unfortunately, at these large meetings, many farmers initially assumed study team members were vendors seeking a commercial relationship. To overcome this barrier, study team members were careful to identify themselves as university-affiliated researchers whose interest was in farmer health and safety, rather than financial. The study team displayed a professionally designed large-scale color banner at each recruitment event. Similarly, professionally-designed name badges, flyers, and business cards were employed to distinguish study team members from vendors who were often found at commodity and advocacy group meetings where prospective study participants were recruited.

Additionally, the study coordinator selected for this role had experience as a farmer and identified himself as such (when appropriate) to prospective individual and group study participants. Thus, prospective study participants may share the unique farmer identity with the study coordinator, possibly favorably influencing their decision to engage in the study.

## Results

For the farm operator intervention study, power analysis initially estimated a sample size of 709 subjects, using an anticipated attrition rate of 40% over the 12-month study period. However, the study team was able to reduce this number after participation records showed that retention of study subjects exceeded anticipated rates. Consequently, the new lower attrition rates resulted in a 30% reduction in target enrollment rates and accompanying lower project costs. The 12-month participant retention rate for the study was 92% (17).

Regarding the farm youth study, a total of 1979 youth were enrolled at 36 sites distributed across the 3 study arms. The retention rate was 29% after 12 months.

## Discussion

As critical as community engagement is to community-based research and the advancement of health promotion goals, there exist multiple obstacles in the context of working with farmers, farm families, and rural populations. The author showcases a variety of community engagement methods employed in two related intervention studies. These community engagement methods were successful in recruiting and retaining hard-to-reach study participants for the studies, and are consistent with those recommended by Poongothai et al. (19).

The study team addressed these barriers through a variety of communication and other methods to optimize community engagement in the interest of enhancing intervention effectiveness. Member participation occurred in virtually all phases of the intervention (e.g., identification of the health problem, design and planning of the intervention, its delivery and its evaluation), and community members served as leaders, collaborators, consultants, informants, and information sources (2–4).

While there is no universal measure of community engagement, study participant enrollment and retention rates can be used. The participant retention rates for the farm operator study cited here was an impressive 92% (17). This rate far exceeds usual retention rates for long-term clinical trials (19). For the farm youth study (18), the retention rate was significantly lower (29%). The reasons for this difference are multiple. Follow-up contacts with youth were more complicated in that the study team member had to first contact the parent, and with permission, was then referred on to the youth, if available and interested. Additionally, there was more mobility in the youth population, with many families moving out of the area of the intervention and not available for follow-up.

The team was strongly committed to the premise that the desired health outcomes for which the studies were designed could occur only when a high level of community engagement was achieved. Because of this, the research team was particularly diligent in applying strategies that would be effective in optimizing community engagement.

The studies highlighted here were limited to English-speaking participants and included persons who self-identified as currently active farm operators or who participated as fourth graders in a rural health and safety education program, most of whom were residential (non-migrant) youth. The aims of the studies highlighted did not include an evaluation of community engagement methods. Since multiple methods were used, and not evaluated individually, the researchers cannot draw conclusions about which methods contributed to the outcomes. Particularly in an era when funding for community-based health research is severely constrained, this is an area ripe for future exploration.

The methods highlighted are highly adaptable to other studies, participants, settings, and health issues, and have potential for augmenting the community engagement and success of future community-based health behavior studies, and consequently contribute to the public health.

## Data Availability

The data supporting the conclusions of this article are available upon request to the corresponding author.

## References

[1] Block P. Community: The structure of belonging. San Francisco: Berrett-Koehler Publishers (2018).

[2] O’Mara-Eves A Brunton G McDaid D Oliver S Kavanagh J Jamal F. Community engagement to reduce inequalities in health: a systematic review, meta-analysis and economic analysis. Public Health Res. (2013) 1. doi: 10.3310/phr01040 25642563

[3] Jain M Shisler S Lane C Bagai A Brown E Engelbert M. Use of community engagement interventions to improve child immunization in low-income and middle-income countries: a systematic review and meta-analysis. BMJ Open. (2022) 12:e061568. doi: 10.1136/bmjopen-2022-061568, PMID: 36351718 PMC9644342

[4] Xie YJ Liao X Lin M Yang L Cheung K Zhang Q. Community engagement in vaccination promotion: systematic review and meta-analysis. JMIR Public Health Surveill. (2024) 10:e49695. doi: 10.2196/49695, PMID: 38478914 PMC11127135

[5] Heo M. Impact of subject attrition on sample size determinations for longitudinal cluster randomized clinical trials. J Biopharm Stat. (2014) 24:507–22. doi: 10.1080/10543406.2014.888442, PMID: 24697555 PMC4034392

[6] McCullagh M Lusk SL Ronis DL. Factors influencing use of hearing protection among farmers: a test of the Pender health promotion model. Nurs Res. (2002) 51:33–9. doi: 10.1097/00006199-200201000-00006, PMID: 11822567

[7] McCullagh M Robertson C. Too late smart: farmers’ adoption of self-protective behaviors in response to exposure to hazardous noise. AAOHN J. (2009) 57:99–105. doi: 10.3928/08910162-20090301-06, PMID: 19338259

[8] McCullagh MC. Effects of a low intensity intervention to increase hearing protector use among noise-exposed workers. Am J Ind Med. (2011) 54:210–5. doi: 10.1002/ajim.20884, PMID: 20721900

[9] National Institute for Occupational Safety and Health. (2007). Have You Heard? Hearing Loss Caused by Noise Is Preventable, 2009.

[10] US Department of Agriculture National Agricultural Statistics Service. (2021). “Family-owned farms account for 96% of U.S. farms, according to the census of agriculture typology report”, News release: Family-owned farms account for 96% of U.S. farms, according to the census of agriculture typology report, January.

[11] US Bureau of Labor Statistics (2025). Union Members Summary. Available online at: http://www.bls.gov/news.release/union2.nr0.htm (Accessed September 5, 2025).

[12] US Bureau of Labor Statistics (2025). “Farmers, Ranchers, and Other Agricultural Managers.” Available online at: https://www.bls.gov/Ooh/Management/Farmers-Ranchers-and-Other-Agricultural-Managers.Htm#tab-5 (Accessed September 5, 2025).

[13] Wilt M. (2025). Rural American mindset, Country Life.

[14] Liebman AK Wiggins MF Fraser C Levin J Sidebottom J Arcury TA. Occupational health policy and immigrant workers in the agriculture, forestry, and fishing sector. Am J Ind Med. (2013) 56:975–84. doi: 10.1002/ajim.22190, PMID: 23606108

[15] Shaghaghi A B.R.S.A. Approaches to recruiting ‘hard-to-reach’ populations into research: a review of the literature. Health Promot Perspect. (2011) 1:86–94. doi: 10.5681/hpp.2011.009, PMID: 24688904 PMC3963617

[16] Garside J Stephenson J Curtis H Morrell M Dearnley C Astin F. Are noise reduction interventions effective in adult ward settings? A systematic review and meta-analysis. Appl Nurs Res. (2018) 44:6–17. doi: 10.1016/j.apnr.2018.08.004, PMID: 30389061

[17] McCullagh MC Banerjee T Cohen MA Yang JJ. Effects of interventions on use of hearing protectors among farm operators: a randomized controlled trial. Int J Audiol. (2016) 55:S3–S12. doi: 10.3109/14992027.2015.1122239, PMID: 26766172 PMC4740201

[18] McCullagh MC Yang JJ Cohen MA. Community-based program to increase use of hearing conservation practices among farm and rural youth: a cluster randomized trial of effectiveness. BMC Public Health. (2020) 20:847. doi: 10.1186/s12889-020-08972-3, PMID: 32493434 PMC7268739

[19] Poongothai S Anjana RM Aarthy R Unnikrishnan R Venkat Narayan KM Ali MK. Strategies for participant retention in long term clinical trials. Perspect Clin Res. (2023) 14:3–9. doi: 10.4103/picr.picr_161_21, PMID: 36909219 PMC10003583

